# Low carbohydrate diets improve atherogenic dyslipidemia even in the absence of weight loss

**DOI:** 10.1186/1743-7075-3-24

**Published:** 2006-06-21

**Authors:** Richard D Feinman, Jeff S Volek

**Affiliations:** 1 Department of Biochemistry, SUNY Downstate Medical Center, Brooklyn, NY 11203, USA; 2 Human Performance Laboratory, Department of Kinesiology, University of Connecticut, Storrs, CT 06269-1110, USA

## Abstract

Because of its effect on insulin, carbohydrate restriction is one of the obvious dietary choices for weight reduction and diabetes. Such interventions generally lead to higher levels of dietary fat than official recommendations and have long been criticized because of potential effects on cardiovascular risk although many literature reports have shown that they are actually protective even in the absence of weight loss. A recent report of Krauss et al. (AJCN, 2006) separates the effects of weight loss and carbohydrate restriction. They clearly confirm that carbohydrate restriction leads to an improvement in atherogenic lipid states in the absence of weight loss or in the presence of higher saturated fat. In distinction, low fat diets seem to require weight loss for effective improvement in atherogenic dyslipidemia.

## Background

The recent report of Krauss et al. [1] highlights the rather dramatic differences in the effects of carbohydrate restricted (CR) and low fat (LF) diets on the lipid changes that may predispose to atherosclerosis. By first implementing weight maintenance diets of different compositions followed by calorie reduction, the authors show that significant improvement in atherogenic lipid states (lower TAG, higher HDL, lower apoB/apoA-1) can be brought about by CR even in the absence of weight loss or in the presence of higher saturated fat. When weight loss was further implemented in the CR groups, there was little further improvement in most markers although HDL continued to increase on calorie reduction. The LF diet, in distinction, required weight loss for effective improvement in the lipid profile, and the additive outcome of diet change and calorie reduction were not as effective as in the CR diets. These results have obvious implications for choice of diets and represents a philosophical reversal of the practical implications of macronutrient composition. Criticism of the use of CR for weight loss has traditionally focused on the potential effect on risk of CVD because of the substitution of fat for carbohydrate. It is now clear that such a change is beneficial and the demonstration that actual weight loss is not required for the benefits of CR suggests the need for reevaluation of current guidelines.

In general, reports on the effects of CR diets continue to defy conventional wisdom. While most official agencies recommend limiting dietary fat or at least saturated fat, experimental data show that replacement of fat, even saturated fat with carbohydrate is deleterious to markers for atherogenic dyslipidemia [2]. Although several studies in the literature had pointed to the value of replacing dietary carbohydrate with fat or protein [3-6], an historical turning point in the reappraisal of CR diets might be considered the study of Foster et al. [7] comparing the Atkins diet with a LF diet. The results were unexpected in that CR, which was anticipated to be deleterious was, in fact, beneficial compared to fat reduction. Studies of CR continue to show improvements in atherogenic dyslipidemia [5,8,9] and there is an evolving picture that the effects of CR, notably in lowering insulin and thereby changing metabolic regulation of lipid, may be more important than the total amount of lipid substrate.

## Atherogenic dyslipidemia

The level of LDL cholesterol is generally considered the most clinically useful marker for cardiovascular disease (CVD) and there seems little question that reduction in LDL, especially if effected by administration of statins, is accompanied by substantial reduction in the risk of cardiovascular events. The importance of LDL as a primary marker, however, must be tempered by observations that LDL is not homogeneous and atherogenicity appears to be a function of particle size: small dense LDL particles are more atherogenic [10]. In addition, other risk factors, high triglyceride (TAG), low HDL, and insulin resistance are frequently increased under conditions that lower LDL [8]. The atherogenicity of a greater number of small LDL particles is reflected in an increase in apolipoprotein B (apoB) since each atherogenic lipoprotein particle contains one molecule of apoB and total LDL would bias results towards lower risk. Barter et al. [11] summarized evidence that apoB is a more reliable indicator of risk than LDL and further that the apoB/apoA-I ratio is superior to conventional cholesterol ratios. Finally, circulating TAG is of considerable importance in this discussion because it is mechanistically linked to the formation of atherogenic particles, and is highly sensitive to dietary manipulation.

## Study synopsis

Details of the dietary intervention of Krauss et al. [1] are shown in Table 1. Diets were consumed over 3 sequential periods in which energy was prescribed to achieve weight maintenance (3 wk), weight loss (-1000 kcal/day; 5 wk), and weight stabilization (4 wk). Blood measures were reported at baseline, after weight maintenance and after weight loss + stabilization. For comparison, the protocol from Sharman, et al. [12] for six weeks on a very low carbohydrate ketogenic diet (VLCKD) designed for weight maintenance are also shown in Table 1.

**Table 1:** Macronutrient composition of diet.  Diet composition from the indicated references.

Group	% CHO	% FAT	% Protein	% SF
Krauss – Ref. [1]				
LF	54	30	16	7
CR (39 % CHO)	39	29	29	8
CR (26 % CHO)	26	46	29	9
CR (26 % CHO + SF)	26	45	29	15
				
Sharman – Ref [12]				
Low Carbohydrate Ketogenic (LCKD)	8	61	30	11
LF (Control)	59	25	15	8

**Figure 1:**
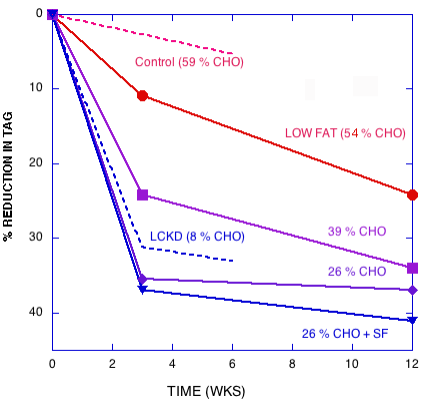
**Effect of dietary interventions on reduction in TAG**. Solid Lines : Data from Reference [1] was converted from reported log values in their Table 2 and per cent of baseline calculated. At week 3, a 1000 kcal reduction in energy was implemented and at week 9, dieters were put on maintenance diet. Combined effect of calorie reduction and maintenance are reported at week 12. Dashed line: Data from reference [12]: A eucaloric ketogenic diet was instituted for six weeks (no weight loss phase). Points were recorded at week 3 and 6.

**Figure 2:**
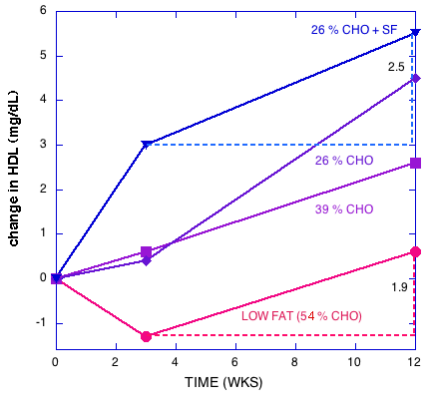
**Change in HDL with diet**. Data from reference [1]. Extrapolated lines are drawn to indicate that there is a greater change during the weight loss phase on low carbohydrate diets with or without saturated fat than on the low fat diet, that is, carbohydrate restriction improves HDL during the macronutrient change and also additively during weight loss.

**Figure 3:**
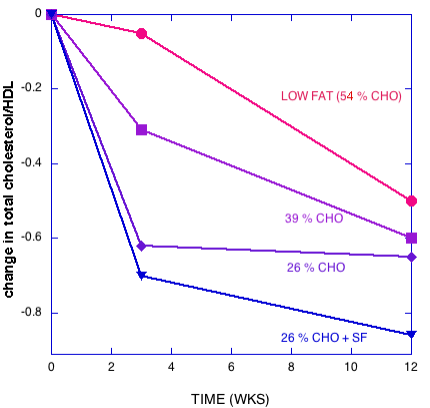
**Change in the ratio of total cholesterol to HDL with diet**. The effect is largely due to HDL increases (Figure 2) since the total changes in LDL were -11.5, -1.8, -6.9 for decreasing carbohydrate. LDL for 26% CHO + SF changed little (+0.4).

**Figure 4:**
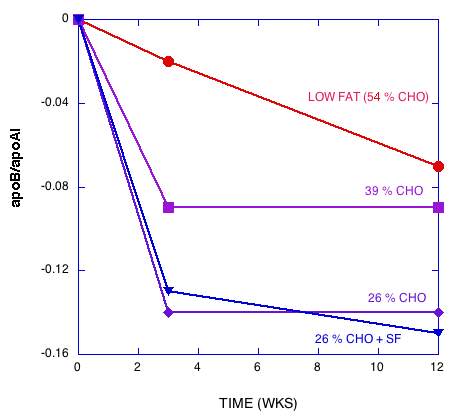
Change in the ratio of apoB:apoA-1 with diet.

**Figure 5:**
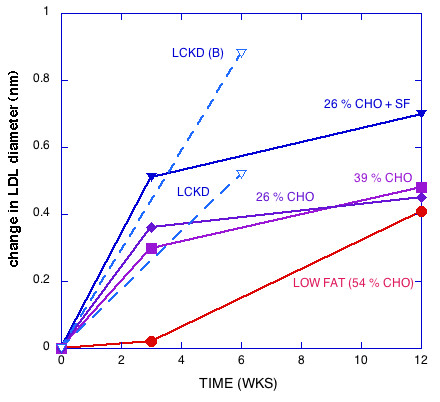
**Change in peak diameter of LDL particles**. Dotted line are data from reference [12] for the effect of LCKD or the subset of effect of LCKD for the population of subjects with high levels of pattern B (small dense LDL particles).

Figures 1, 2, 3, 4, 5 show the results of sequential change in macronutrient composition and caloric restriction. Overall, the data show a significantly greater improvement in lipid profile as carbohydrate is reduced even if this change is not accompanied by caloric reduction and even in the presence of relatively high saturated fat, in agreement with results of Sharman, et al. [12]. Data from Sharman's study is indicated by dotted lines in Figures 2 and 5. When caloric restriction is introduced after these weight stable changes, there is little additional change in most parameters on the CR diets, but the additive effects of the CR and caloric reduction are greater than the additive effects of fat reduction followed by caloric restriction (e.g., compare week Wk 12 values for the 56% CHO to the 26% CHO + SF diets in Figures 1, 2, 3, 4, 5).

## Mechanisms and the separation of carbohydrate restriction and weight loss

The striking data tabulated by Krauss, et al. [1] and presented in graphic form in Figures 1, 2, 3, 4, 5 are somewhat at odds with their stated conclusion: "Moderate carbohydrate restriction and weight loss provide equivalent but non-additive approaches to improving atherogenic dyslipidemia" and "the beneficial lipid changes resulting from a reduced carbohydrate intake were not significant after weight loss."

It is not clear what is meant by additive since the combined effects due to macronutrient change and caloric restriction are not compared to experiments where they are implemented together which are known to provide large positive effects. In any case, at least HDL values do show significant increases in both phases of the experiment, positive for low carbohydrates, initially negative and then positive for low fat and there is a pronounced additive effect (Figure 2).

Compared to weight loss on a LF diet, the high saturated fat CR diet with no weight loss resulted in better improvements in LDL peak size, TAG, HDL, and the ratios total cholesterol/HDL and apoB/ApoA-1, that is, the effects are not equivalent; CR is significantly better than weight loss in the presence of LF for atherogenic dyslipidemia. The fact that these effects are not equivalent is further shown in Figures 1, 2, 3, 4, 5, where the combined (weight stable and weight loss) effects for LF are not as great as for CR. The results suggest that if, at week 12, a 26 % CR with saturated fat were instituted for the LF group, further improvement in lipid profile would be brought about.

Krauss et al. [1] make the underlying assumption that weight loss is the same whether caused by caloric restriction in the presence of low carbohydrate or low fat but their data show that this is not so. It seems that they also assume that weight loss is a cause not an effect (due to calorie reduction). Whereas this may play a role, it is reasonable to assume that improvement in dyslipidemia *and *weight loss are parallel *responses *to calorie reduction which is the major physiologic stimulus. It is obvious that the reason lipid changes are not brought about in Krauss's experiment by weight loss in the low carbohydrate arms is that the lipid markers have already changed drastically. Finally, it should be pointed out that the reduction in calories that is effective in the weight loss phase of the low fat arm included a substantial reduction in carbohydrate as one component.

## Summary

Although some effort is required to disentangle the data and interpretation, the recent publication from Krauss et al. [1] should be recognized as a breakthrough. Their findings, presented in Figures 1, 2, 3, 4, 5 make it clear that the salutary effects of CR on dyslipidemia do not require weight loss, a benefit that is not a feature of strategies based on fat reduction. As such, Krauss et al. [1] provides one of the strongest arguments to date for CR as a fundamental approach to diet, especially for treating atherogenic dyslipidemia.

## Abbreviations

apo: apolipoprotein, CR: carbohydrate restricted diet, CVD: cardiovascular disease, HDL: high density lipoprotein cholesterol, LDL: low density lipoprotein cholesterol, LF: low fat diet, SF: saturated fat, TAG: triacylglycerol (triglyceride)

## Competing interests

The author(s) declare that they have no competing interests.
